# Variation of Secondary Metabolite Profile of *Zataria multiflora* Boiss. Populations Linked to Geographic, Climatic, and Edaphic Factors

**DOI:** 10.3389/fpls.2020.00969

**Published:** 2020-07-03

**Authors:** Ali Karimi, Andrea Krähmer, Nadine Herwig, Hartwig Schulz, Javad Hadian, Torsten Meiners

**Affiliations:** ^1^ Institute for Ecological Chemistry, Plant Analysis and Stored Product Protection, Julius Kühn Institute, Berlin, Germany; ^2^ Institute of Pharmacy, Freie Universität Berlin, Berlin, Germany; ^3^ Department of Agriculture, Medicinal Plants and Drug Research Institute, Shahid Beheshti University, Tehran, Iran

**Keywords:** near-infrared spectroscopy, essential oil, carvacrol, linalool, chemical diversity, environmental factors, soil chemistry, *Zataria multiflora* Boiss

## Abstract

Geographic location and connected environmental and edaphic factors like temperature, rainfall, soil type, and composition influence the presence and the total content of specific plant compounds as well as the presence of a certain chemotype. This study evaluated whether geographic, edaphic, and climatic information can be utilized to predict the presence of specific compounds from medicinal or aromatic plants. Furthermore, we tested rapid analytical methods based on near infrared spectroscopy (NIR) coupled with gas chromatography/flame ionization (GC/FID) and gas chromatography/mass spectrometry (GC/MS) analytical methods for characterization and classification metabolite profiling of *Zataria multiflora* Boiss. populations. *Z. multiflora* is an aromatic, perennial plant with interesting pharmacological and biological properties. It is widely dispersed in Iran as well as in Pakistan and Afghanistan. Here, we studied the effect of environmental factors on essential oil (EO) content and the composition and distribution of chemotypes. Our results indicate that this species grows predominantly in areas rich in calcium, iron, potassium, and aluminum, with mean rainfall of 40.46 to 302.72 mm·year^−1^ and mean annual temperature of 14.90°C to 28.80°C. EO content ranged from 2.75% to 5.89%. Carvacrol (10.56–73.31%), thymol (3.51–48.12%), linalool (0.90–55.38%), and *p*-cymene (1.66–13.96%) were the major constituents, which classified 14 populations into three chemotypes. Corresponding to the phytochemical cluster analysis, the hierarchical cluster analysis (HCA) based on NIR data also recognized the carvacrol, thymol, and linalool chemotypes. Hence, NIR has the potential to be applied as a useful tool to determine rapidly the chemotypes of *Z. multiflora* and similar herbs. EO and EO constituent content correlated with different geographic location, climate, and edaphic factors. The structural equation models (SEMs) approach revealed direct effects of soil factors (texture, phosphor, pH) and mostly indirect effects of latitude and altitude directly affecting, e.g., soil factors. Our approach of identifying environmental predictors for EO content, chemotype or presence of high amounts of specific compounds can help to select regions for sampling plant material with the desired chemical profile for direct use or for breeding.

## Introduction

All over the world, plants face different local climatic regimes as well as different edaphic factors. To predict how different environmental factors affect species dispersal, the abundance of populations and chemotypes as well as the content of specific compounds can be a valuable tool to understand plant variation in chemical features. It can also facilitate prospecting plants with high amounts of specific compounds for nutrition, pharmaceutical or agricultural use. In most cases, plant essential oils (EOs) are characterized by a strong aroma, which is mainly produced by secondary metabolites. EO compounds are coupled with environmental acclimatization and play vital biological roles. Several factors, such as environmental and edaphic conditions, geographical regions, season of collection, harvesting time, genotype, and ecotype influence the quantitative and qualitative composition of EO (Milos et al., 2001; Zgheib et al., 2016; Morshedloo et al., 2018). For example, in *Matricaria chamomilla* L. climatic conditions, altitude, soil properties, and irrigation influence the phytochemical composition and antioxidant activity of EO (Formisano et al., 2015).

**Figure 1:**
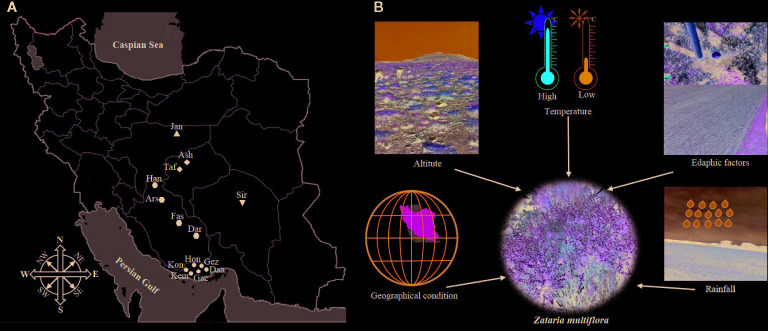
Collection sites **(A)** and overview on geographic, climatic, and edaphic factors **(B)** affecting *Zataria multiflora* populations from Iran.

*Zataria multiflora* Boiss. (Lamiaceae) is an aromatic and perennial shrub growing wild in Iran (**Figure 1A**), Pakistan, and Afghanistan. This aromatic plant is known by the Persian name of Avishan Shirazi which is also entitled Sattar or Zattar, meaning thyme. *Z. multiflora* can be identified by the orbicular, densely gland-dotted, grey-green ovate leaves, and the thickly white hairy round buds in the leaf axils. Its inflorescence is verticillate, and the flowers are very small and white (Simbar et al., 2008). *Z. multiflora* has shown pharmacological (antimicrobial, antinociceptive, spasmolytic, and anti-inflammatory) properties, is utilized in traditional folk remedies for its antiseptic, analgesic, carminative, anthelmintic, and antidiarrheal properties, and it is also a condiment (Iranian Herbal Pharmacopoeia Committee, 2002; Moazeni et al., 2014; Khazdair et al., 2018; Mohajeri et al., 2018). Currently, some pharmaceutical forms of this plant, such as syrups, oral drops, soft capsules, and vaginal creams are produced (Sajed et al., 2013; Mahboubi, 2019).

The EO of *Z. multiflora* is rich in phenolic oxygenated monoterpenes. The main chemical constituents are carvacrol, thymol, linalool, and *p*-cymene (Hadian et al., 2011a; Saedi Dezaki et al., 2016; Mahmoudvand et al., 2017). Although there are some studies based on *Z. multiflora* EO constituents (Saleem et al., 2004; Niczad et al., 2019), there is hardly any information on the environmental factors affecting EO content and composition. *Z. multiflora* is not only harvested for local markets but is also one of the valuable species for industry, so this plant is under severe threat from overharvesting. Thus, a deep perception of its phytochemical and environmental characteristics in its natural habitats is crucial to foretell its behavior under man-made cultivation.

Today, the standard method for EO analysis is gas chromatography coupled with different detection techniques like mass spectrometry. In the last two decades, numerous vibrational spectroscopy methods including mid-infrared (IR), near-infrared (NIR), and Raman spectroscopy have been described as a useful tool to examine the plant secondary metabolites which are commonly applied in the chemical fingerprinting of plants (Schulz et al., 2004; Schulz et al., 2005; Gudi et al., 2014). However, up to now, no studies have been performed utilizing this capable approach to differentiate and characterize various *Z. multiflora* chemotypes.

The aim of this study was to evaluate how different environmental factors affect species dispersal with respect to EO production, chemotype as well as the content of specific compounds of *Z. multiflora* population (**Figures 1A, B**). Besides, we aimed to evaluate whether geographic, edaphic, and climatic information can predict the presence of specific compounds. Furthermore, we tested rapid analytical methods based on NIRS coupled with GC/GC-MS methods for characterization and classification metabolite profiling of *Z. multiflora* populations.

## Materials and Methods

### Study Area

To determine the effects of geography, climate, and edaphic conditions on EO yield and composition of *Z. multiflora*, plant materials were collected in 2018 in 14 natural habitats across five provinces from the center to the south of Iran including their major growing areas Isfahan, Kerman, Yazd, Fars, and Hormozgan provinces (**Figure 1A**).

### Plant Material and Chemicals

Plant samples were collected in June 2018 at the flowering stage. At each region, 6 to 11 individual shrubs were collected depending on the population size with a minimum distance of 100 m. Voucher specimens (no. MPH-1799) were authenticated and deposited in the Herbarium of Medicinal Plants and Drugs Research Institute (MPH), Shahid Beheshti University, Tehran, Iran. Geographical data and altitude for each sampling area were recorded using GPS (**Table 1**). Besides, climate data for five years were taken from metrological stations closest to the habitats (**Table 2**). Carvacrol, linalool, *p*-cymene, and *γ*-terpinene were purchased from Sigma-Aldrich-Fluka (Germany), and thymol and *α*-pinene from Roth (Germany).

**Table 1:** General information on natural habitats of *Zataria multiflora* populations.

Population name	Code	Province	Location	Latitude (N)	Longitude (E)	Altitude (m)
Jandaq	Jan	Esfahan	Jandaq toward mesr desert	33° 57' 44''	54° 31' 02''	1235
Ashkezar	Ash	Yazd	Zarband village	31° 48' 49''	54° 00' 26''	1946
Taft	Taf	Yazd	Darreh-ye Gahan mountains	31° 42' 26''	54° 10'	1697
Siriz	Sir	Kerman	Hamsij village	30° 55' 43''	55° 57' 01''	1763
Fasa	Fas	Fars	Kohankouye village	28° 59' 27''	53° 42' 25''	1516
Arsenjan	Ars	Fars	Tange laykhare mountains	29° 53' 49''	53° 16' 20''	1865
Haneshk	Han	Fars	Haneshk village, Safashahr	30° 49' 16''	53° 18' 19''	1898
Darab	Dar	Fars	Tange Talar Jangi mountains	28° 44' 27''	54° 34' 41''	1276
Gezeh	Gez	Hormozgan	Cheshmeh-ye seyyed	27° 06' 35''	54° 04' 46''	731
Hongooyeh	Hon	Hormozgan	Darreh-ye Baraveh	27° 06' 19''	54° 04' 07''	820
Daarbast	Daa	Hormozgan	Daarbast	26° 58' 02''	54° 01' 59''	1009
Gachooyeh	Gac	Hormozgan	Gachooyeh	26° 58' 28''	53° 58' 06''	1055
Kemeshk	Kem	Hormozgan	Kemeshk	27° 03' 13''	53° 50' 41''	937
Konar Siah	Kon	Hormozgan	Konar Siah	27° 09' 05''	53° 57' 04''	981

**Table 2:** Edaphic factors and climatic characteristics in natural habitats of *Zataria multiflora*.

Region	Rainfall [mm year^−1^]	M-Temp [°C]	Soil texture	pH	OM N		P K Ca F Al				
					[%]		[mg kg^−1^]				
Jandaq	55.86	21.50	Silty sand	7.70	4.43	0.06	394.30	5581.40	69376.20	16424.20	17012.60
Ashkezar	40.46	21.10	Loamy sand	7.80	5.17	0.04	409.70	7171.40	155569.40	14486.10	20489.70
Taft	49.76	20.30	Sandy loamy silt	7.70	5.74	0.04	354.90	7925.10	112373.80	19637.10	20137.30
Siriz	107.80	20.20	Sandy loam	7.70	4.44	0.03	416.90	6863.10	75182.10	20339.10	20521.30
Fasa	278.48	20.30	Clayey loam	7.60	6.97	0.06	329.20	8441.60	96355.40	23471.60	27656.70
Arsenjan	215.22	20.30	Sandy loamy silt	7.70	7.54	0.18	346.20	6151.10	117935.10	20298.30	20936.10
Haneshk	180.06	14.90	Loamy sand	7.60	4.00	0.09	342.60	12427.10	12756.10	24538.40	34280.10
Darab	276.38	24.30	Silty loam	7.60	10.0	0.23	312.60	5306.80	116218.30	16820.20	18424.30
Gezeh	302.72	28.80	Sandy loam	7.80	7.02	0.05	293.40	3609.80	174266.60	11979.30	12212.20
Hongooyeh	302.72	28.80	Silty loamy sand	7.90	7.54	0.03	355.90	3640.50	139953.40	14064.00	12378.00
Daarbast	302.72	28.80	Sandy loam	7.80	6.59	0.03	232.80	1993.80	197820.30	8190.60	6945.90
Gachooyeh	302.72	28.80	Loamy sand	7.80	7.02	0.03	222.50	2368.80	195214.40	9272.40	7986.60
Konar Siah	302.72	28.80	Loamy sand	7.80	7.02	0.05	275.80	2272.10	178396.30	7442.80	6327.50
Kemeshk	302.72	28.80	Loamy sand	7.80	7.02	0.04	249.20	2320.40	186805.30	8357.60	7157.10

M-Temp, Mean annual temperature; OM, organic matter.

### Soil Analysis

Soil samples from the surface layer (0 to 30 cm depth) were taken from five randomly selected plots in each sampling site. The five soil samples were combined into a single 500 g sample that was dried at room temperature (20–25°C) and sieved to 2 mm. A duplicate soil sample was sieved through a 2 mm filter once again for determination of soil chemical characteristics including the soil texture (percentage content of sand, silt, and clay), the amount of abundant nutrients (N, P, K, Ca, Al, and Fe), pH value, and organic matter. The total heavy metal and nutrient contents of soil samples were determined after pressure dissolution with 69% supra pure nitric acid (according to A2.4.3.1, VDLUFA, 1991) by ICP-AES (iCAP™ 7600 Duo, Thermo Fischer Scientific). Contents of total carbon and total nitrogen were determined with CNS elemental analyzer (Vario EL Cube, Elementar Analysesysteme GmbH). Pedological base parameters (soil particle size, pH value, C/N) were collected for characterization. The particle size determination of soil texture was performed according to DIN 19683-2 (1997).

### Isolation of the Essential Oils

The aerial plant parts were dried at room temperature (20–25°C) in the shade, then the leaves of each plant were separated and 10 g of each plant sample were ground manually. The EO of each sampled plant (10 g of leaves) was isolated by hydro-distillation for 2 h utilizing a clevenger-type system (Pavela et al., 2018). The distilled oils were dried over anhydrous sodium sulfate and stored at 4°C in sealed glass vials for analysis. The yield of the essential oil was calculated based on the dry weight of the plant material.

### GC-FID and GC/MS Analyses

EOs were analyzed by GC−FID using an Agilent gas chromatograph 6890N, equipped with a HP-5 column (30 m × 0. 25 mm i.d., with a film thickness of 0.5 μm). The oven temperature was programmed at 50°C for 2 min, then from 50°C to 320°C at 5°C min^−1^, and held at 320°C for 6 min. Both injector and detector temperatures were 250°C. Hydrogen was used as carrier gas with a constant flow rate of 1 ml min^−1^, and 1 μl of the diluted EOs (1/500 v/v in isooctane) was injected automatically (Gerstel MPS) in a splitless mode. Nitrogen was used as make-up gas, which was set at a flow of 45 ml min^−1^.

Mass spectrometry of the EOs was performed using an Agilent MSD 5975B/GC 6890N, equipped with a 30 m × 0.25 mm i.d., 0.5 μm, HP-5MS column. The injector temperature was 250°C, and the initial GC oven temperature was 50°C, held for 2 min, then raised to 320°C at 5°C min^−1^ and held for 6 min. Helium was used as carrier gas with a flow rate of 1 ml min^−1^. One μl of the diluted EOs (1/500 v/v in isooctane) was injected automatically (Gerstel MPS) in a splitless mode. Injector and detector temperatures were set at 250°C. The EI^+^-MS operating parameters were as follows: ionization energy, 70 eV and ion source temperature, 230°C. The quadrupole mass spectrometer was scanned over 35 to 350 *m/z*. The runtime and solvent delay were set at 60 and 5 min, respectively (4.45 scans/s). Carvacrol, thymol, linalool, *p*-cymene, *γ*-terpinene, and *α*-pinene were used as standard. 6-Methyl-5-hepten-2-one was used as internal standard and was added to the dilution before the analysis. The oil components were identified by comparison of mass spectra and retention indices with those recorded in the Adams (Adams, 2014), NIST mass spectral databases SRD 69 (NIST Chemistry WebBook, 2002), standard constituents, and the previously published data. The retention indices of individual components were calculated using a series of n-alkanes (C8-C40) (Sigma-Aldrich-Fluka, Germany) (1/100 in n-Pentan). The relative percentage composition of individual compounds was computed from the GC peak areas obtained without using correction factors.

### NIR Spectroscopy and Chemometrics

Before isolation of EO, vibrational spectroscopy was performed directly on the homogenized plant material. NIRS analyses were carried out on a Fourier-Transform (FT)-NIR spectrometer (Multi-Purpose Analyser MPA, Bruker Optics GmbH, Germany). Spectra were recorded in the wavenumber range of 4,000 to 12,000 cm^−1^ with a spectral resolution of 8 cm^−1^. Approximately 7 g of dried leaves were put in a glass Petri dish and spectra were collected during rotation of the dish using the integrating sphere for measuring in diffuse reflection. Spectra were acquired at 30 s. Each sample was analyzed with threefold repetition. The raw spectra were centered and corrected for scattering effects and baseline shifting using WMSC of the OPUS 6.5 software (Bruker Optics). Only averaged spectra of the three replicates were used for the later chemometric analysis.

### Statistical Analysis

Statistical analysis was performed using hierarchical cluster analysis (HCA) with SPSS version 16 to classify and cluster the populations of *Z. multiflora* based on the squared Euclidean distances. Pearson’s correlation coefficients were estimated among the EO content, major components, and edaphic factors using SPSS (SPSS, Chicago, IL, USA) software package from version 16. The calculation of means, standard deviations (SD) and t-test were used to express the significance of differences (P < 0.05) using SAS 9.1 program (SAS Inc. USA).

For chemometrics (based on NIR), HCA was performed to evaluate the diversity of the samples. Characteristic spectral ranges were identified by comparison with spectra appropriate reference standards and HCA. Calibration models were built by 10-fold cross-validation using a partial least squares (PLS) algorithm. Therefore, GC data of each plant and averaged plant wise spectra of the population were correlated.

Furthermore, we set up SEMs for each region using partial least squares (PLS) regression using Warp PLS 6.0 (Kock and Lynn, 2012). The PLS regression was chosen over covariance based approaches because it suited our small sample size and, compared to covariance structure analysis, can accommodate both reflective and formative scales more easily. Moreover, PLS does not require any *a priori* distributional assumptions (Chin and Newsted, 1999). We present individual standardized path coefficients (β), partial model fit scores (R^2^), and overall model P values calculated by resampling estimations coupled with Bonferroni like corrections (Kock, 2010). To validate the models three model-fit indices [average path coefficient (APC), average R-squared (ARS), and average variance inflation factor (AVIF)] were calculated for each region. For model fit, it is recommended that P values for APC and ARS are both lower than 0.05 (i.e., significance at the 0.05 level). The AVIF index controls for multicollinearity and should be below 5 (Kock, 2010). In the SEM analysis we set paths from geographic factors (latitude, longitude, altitude), climatic factors (rainfall, temperature), soil texture (relative proportion of clay, silt, and sand), constituents (N, P, K, Al, Ca, Fe), and pH value directly to EO content and compounds; furthermore, we included the possible effects of the geographic factors on climatic and soil factors.

## Results

### Phytochemical Analysis of Essential Oil

**Figure 2:**
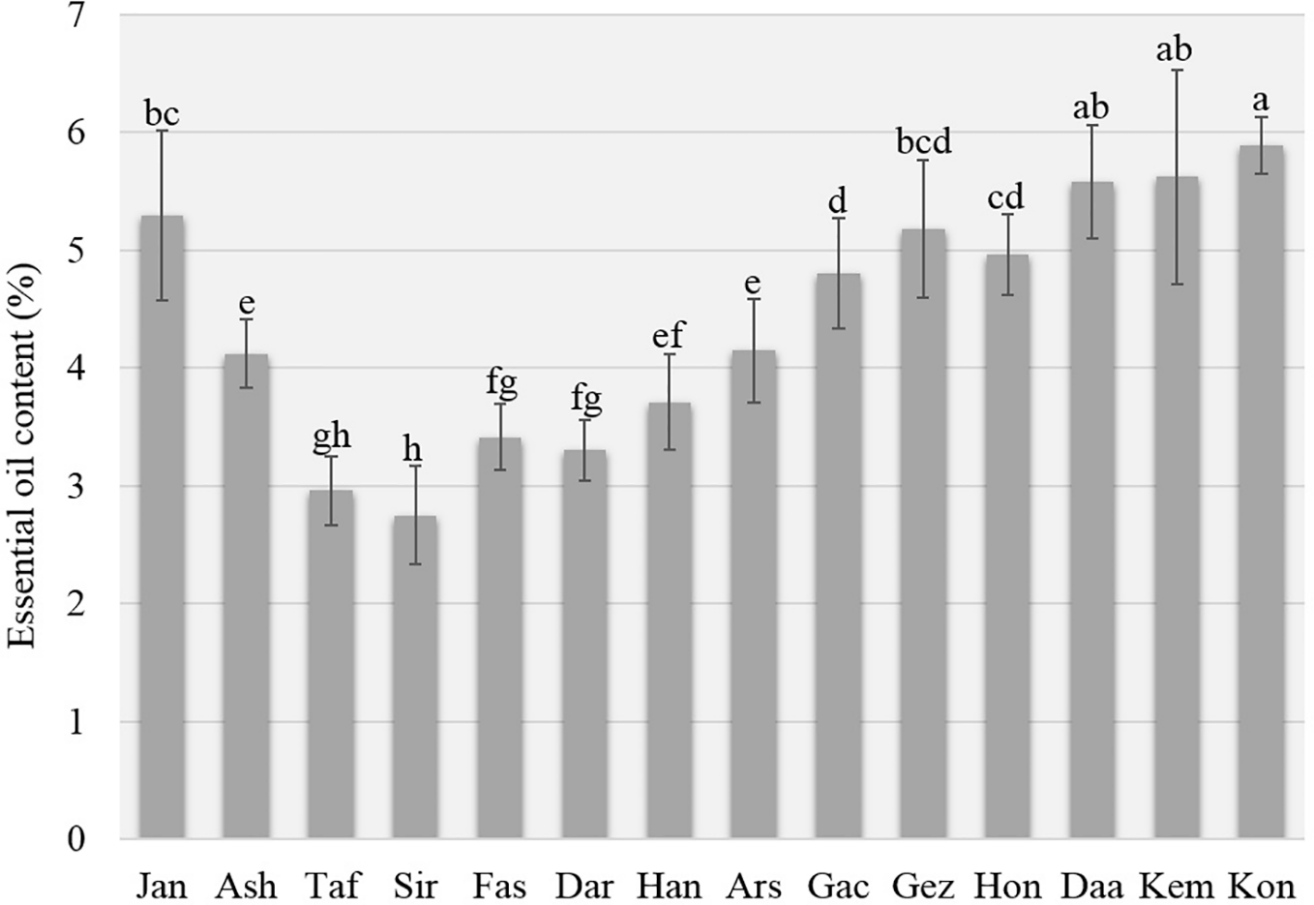
Essential oil content of *Zataria multiflora* populations.

The EOs were obtained and analyzed by hydro-distillation and GC-FID/GC-MS respectively. There was a significant difference in EO content among the studied populations. The EO content ranged from 2.75 (for population Siriz) to 5.89% in dry matter (DM), (for population Konar Siah) (**Figure 2**). Fifty-six compounds were identified with significant differences between the populations (**Table 3**). The oils mainly consisted of carvacrol (10.56–73.31%), thymol (3.51–48.12%), linalool (0.90–55.38%), *p*-cymene (1.66–13.96%), *γ*-terpinene (0.99–6.28%), *α*-pinene (0.93–4.01%), carvacrol methyl ether (0.39–3.71%), myrcene (0.94–2.77%), *E*-caryophyllene (1.09–2.37%), and *α*-terpinene (0.39–1.61%).

Table 3Variation of the phytochemical compositions (%) among the studied populations of *Zataria multiflora*.

**Table:** 

N	Components	RI^a^	RI^b^	Populations					
				Jandaq	Ashkezar	Taft	Siriz	Fasa	Methods
1	*α*-Thujene	933	929	0.43 ± 0.06	0.18 ± 0.10	0.41 ± 0.03	0.14 ± 0.05	0.30 ± 0.03	RI, MS
2	*α*-Pinene	940	939	0.93 ± 0.28	1.82 ± 0.23	2.01 ± 0.14	1.85 ± 0.33	1.64 ± 0.18	RI, MS
3	Camphene	955	954	0.10 ± 0.03	0.12 ± 0.01	0.12 ± 0.01	0.10 ± 0.01	0.10 ± 0.01	RI, MS
4	2,4(10)-Thujadiene	960	957	–	–	–	tr	–	RI, MS
5	1,3-Octanol	969	973	0.07 ± 0.01	0.07 ± 0.01	0.07 ± 0.01	0.09 ± 0.01	0.08 ± 0.01	RI, MS
6	Sabinene	979	977	0.11 ± 0.01	0.07 ± 0.01	0.11 ± 0.01	0.09 ± 0.02	0.08 ± 0.01	RI, MS
7	*β*-Pinene	983	978	0.23 ± 0.03	0.28 ± 0.03	0.37 ± 0.01	0.34 ± 0.06	0.33 ± 0.01	RI, MS
8	Myrcene	995	992	1.35 ± 0.51	1.15 ± 0.16	1.39 ± 0.14	2.77 ± 0.51	1.45 ± 0.17	RI, MS
9	3-Octanol	997	995	0.05 ± 0.01	0.06 ± 0.01	0.10 ± 0.05	0.21 ± 0.04	0.10 ± 0.06	RI, MS
10	*α*-Phellandrene	1010	1005	0.15 ± 0.04	0.17 ± 0.01	0.19 ± 0.01	0.29 ± 0.03	0.21 ± 0.01	RI, MS
11	*δ*-3-Carene	1015	1011	0.02 ± 0.01	0.04 ± 0.02	0.02 ± 0.01	0.03 ± 0.01	–	RI, MS
12	*α*-Terpinene	1021	1019	0.92 ± 0.08	1.00 ± 0.07	1.38 ± 0.26	0.39 ± 0.14	1.61 ± 0.09	RI, MS
13	*p*-Cymene	1030	1025	5.88 ± 1.38	5.69 ± 0.31	7.29 ± 1.17	1.66 ± 0.95	7.21 ± 0.42	RI, MS
14	Limonene	1034	1030	0.37 ± 0.10	0.49 ± 0.07	0.54 ± 0.12	0.81 ± 0.07	0.61 ± 0.03	RI, MS
15	1,8-Cineole	1037	1035	0.24 ± 0.10	0.05 ± 0.01	0.08 ± 0.01	0.46 ± 0.10	0.03 ± 0.01	RI, MS
16	*Z*-*β*-Ocimene	1039	1039	0.28 ± 0.08	0.41 ± 0.04	0.44 ± 0.10	0.69 ± 0.21	0.37 ± 0.05	RI, MS
17	*E*-*β*-Ocimene	1050	1050	0.15 ± 0.10	0.07 ± 0.01	0.12 ± 0.04	1.31 ± 0.12	0.28 ± 0.05	RI, MS
18	*γ*-Terpinene	1063	1062	3.65 ± 0.72	3.14 ± 0.26	5.24 ± 1.12	0.99 ± 0.57	6.28 ± 0.40	RI, MS
19	*Z*-Sabinene hydrate	1071	1070	0.30 ± 0.01	0.14 ± 0.06	0.27 ± 0.03	0.08 ± 0.02	0.18 ± 0.01	RI, MS
20	*Z*-Linalool oxide	1076	1074	0.04 ± 0.01	0.02 ± 0.01	0.05 ± 0.01	1.67 ± 0.57	0.15 ± 0.03	RI, MS
21	Terpinolene	1092	1089	0.22 ± 0.09	0.21 ± 0.03	0.27 ± 0.10	1.62 ± 0.42	0.35 ± 0.03	RI, MS
22	Linalool	1103	1100	1.19 ± 0.23	1.62 ± 0.60	3.35 ± 2.20	55.38 ± 6.4	9.59 ± 1.83	RI, MS
23	*E*-*γ*-Caryophyllene	1106	1106	0.11 ± 0.04	0.04 ± 0.01	0.06 ± 0.04	1.37 ± 0.17	0.27 ± 0.05	RI, MS
24	1-Octenyl-3-acetate	1111	1113	–	–	–	0.20 ± 0.10	–	RI, MS
25	*p*-Menth-2-en-1-ol	1124	1122	–	–	–	0.32 ± 0.18	–	RI, MS
26	allo-Ocimene	1131	1132	–	–	–	0.16 ± 0.11	0.11 ± 0.02	RI, MS
27	1,3,8-*p*-Menthatriene	1133		–	–	–	0.12 ± 0.01	–	MS
28	Borneol	1172	1171	tr	–	0.02 ± 0.01	0.63 ± 0.18	0.21 ± 0.04	RI, MS
29	*Z*-Linalool oxide (pyranoid)	1177	1173	0.10 ± 0.01	0.02 ± 0.01	0.05 ± 0.02	0.10 ± 0.01	0.08 ± 0.01	RI, MS
30	4-Terpineol	1183	1179	0.09 ± 0.01	0.14 ± 0.03	0.14 ± 0.01	0.25 ± 0.03	0.15 ± 0.01	RI, MS
31	*p*-Cymenol-8	1188	1184	0.46 ± 0.04	0.46 ± 0.02	0.49 ± 0.04	0.23 ± 0.03	0.43 ± 0.02	RI, MS
32	*α*-Terpineol	1195	1190	0.44 ± 0.13	0.56 ± 0.05	0.56 ± 0.11	0.68 ± 0.05	0.45 ± 0.02	RI, MS
33	*Z*-Dihydro carvone	1202	1200	0.15 ± 0.01	0.17 ± 0.01	0.10 ± 0.03	0.15 ± 0.06	0.06 ± 0.04	RI, MS
34	Nerol	1234	1228	0.07 ± 0.02	0.06 ± 0.01	–	0.10 ± 0.02	–	RI, MS
35	Thymol methyl ether	1238	1237	–	0.08 ± 0.03	0.35 ± 0.19	0.20 ± 0.08	0.88 ± 0.06	RI, MS
36	Carvacrol methyl ether	1248	1241	1.04 ± 0.37	1.50 ± 0.40	1.00 ± 0.21	0.39 ± 0.10	0.49 ± 0.02	RI, MS
37	Geraniol	1260	1263	0.04 ± 0.01	–	0.06 ± 0.03	1.00 ± 0.43	0.11 ± 0.02	RI, MS
38	Geranial	1280	1273	–	–	–	tr	–	RI, MS
39	*p*-Thymol	1286		0.07 ± 0.03	0.08 ± 0.01	0.10 ± 0.02	0.02 ± 0.01	0.10 ± 0.01	MS
40	Thymol	1295	1295	3.51 ± 3.27	9.94 ± 2.03	25.32 ± 12.7	6.17 ± 3.00	48.12 ± 2.9	RI, MS
41	Carvacrol	1309	1305	73.31 ± 4.3	65.14 ± 1.8	42.23 ± 15.3	10.56 ± 3.9	12.42 ± 2.8	RI, MS
42	Thymol acetate	1358	1359	0.02 ± 0.01	0.10 ± 0.03	0.49 ± 0.27	0.10 ± 0.03	0.87 ± 0.07	RI, MS
43	Carvacrol acetate	1377	1368	0.80 ± 0.21	0.80 ± 0.13	0.60 ± 0.19	0.20 ± 0.07	0.16 ± 0.01	RI, MS
44	*β*-Bourbonene	1384	1378	–	–	–	0.09 ± 0.04	–	RI, MS
45	*E*-Caryophyllene	1435	1427	1.09 ± 0.22	1.61 ± 0.21	2.16 ± 0.42	1.56 ± 0.60	1.85 ± 0.20	RI, MS
46	Aromadendrene	1455	1436	0.29 ± 0.06	0.40 ± 0.07	0.37 ± 0.08	0.14 ± 0.10	0.19 ± 0.05	RI, MS
47	*α*-Humullene	1469	1452	0.10 ± 0.01	0.11 ± 0.01	0.14 ± 0.02	0.14 ± 0.04	0.13 ± 0.01	RI, MS
48	9-epi-(*E*)-Caryophyllene	1477	1474	–	–	–	–	–	RI, MS
49	*E*-*β*-Guaiene	1504	1498	–	–	–	0.02 ± 0.01	–	RI, MS
50	Viridiflorene	1510	1505	0.24 ± 0.10	0.26 ± 0.05	0.28 ± 0.08	0.19 ± 0.10	0.18 ± 0.02	RI, MS
51	Spathulenol	1594	1578	0.38 ± 0.09	0.35 ± 0.03	0.36 ± 0.06	0.59 ± 0.17	0.39 ± 0.02	RI, MS
52	Isoaromadendrene epoxide	1597		0.08 ± 0.01	0.11 ± 0.01	0.12 ± 0.02	0.47 ± 0.09	0.14 ± 0.02	MS
53	Caryophyllene oxide	1601	1599	0.18 ± 0.03	0.20 ± 0.01	0.20 ± 0.05	0.76 ± 0.11	0.21 ± 0.02	RI, MS
54	Caryophylla-4(12),8(13)-dien-5 beta-ol	1654	1663	0.07 ± 0.01	0.08 ± 0.01	0.09 ± 0.01	0.32 ± 0.03	0.11 ± 0.01	RI, MS
55	14-Hydroxy-9-epi-(*E*)-caryophyllene	1673	1669	0.03 ± 0.02	0.07 ± 0.02	0.08 ± 0.01	0.34 ± 0.06	0.09 ± 0.01	RI, MS
56	Khusinol	1686	1674	0.05 ± 0.03	0.13 ± 0.01	0.08 ± 0.01	0.27 ± 0.03	0.09 ± 0.01	RI, MS
	Monoterpene hydrocarbons			14.79	14.84	19.90	13.36	20.93	
	Oxygenated monoterpens			81.87	80.88	75.26	78.89	74.48	
	Sesquiterpene hydrocarbons			1.83	2.42	3.01	3.51	2.62	
	Oxygenated sesquiterpenes			0.80	0.94	0.93	2.75	1.03	
	Essential oil content (%)			5.29 ± 0.72	4.12 ± 0.29	2.96 ± 0.29	2.75 ± 0.42	3.41 ± 0.28

**Table:** 

N	Components	RI^a^	RI^b^	Populations					
				Gachooyeh	Kemeshk	Gezeh	Hongooyeh	Arsenjan	Methods
1	*α*-Thujene	933	929	0.29 ± 0.06	0.27 ± 0.11	0.47 ± 0.13	0.41 ± 0.09	0.33 ± 0.03	RI, MS
2	*α*-Pinene	940	939	2.57 ± 0.74	2.59 ± 0.73	4.01 ± 0.97	3.23 ± 1.15	1.43 ± 0.20	RI, MS
3	Camphene	955	954	0.14 ± 0.02	0.14 ± 0.02	0.11 ± 0.04	0.17 ± 0.04	0.10 ± 0.01	RI, MS
4	2,4(10)-Thujadiene	960	957	–	–	–	–	–	RI, MS
5	1,3-Octanol	969	973	0.07 ± 0.01	0.07 ± 0.01	0.07 ± 0.01	0.08 ± 0.01	0.07 ± 0.01	RI, MS
6	Sabinene	979	977	0.09 ± 0.01	0.09 ± 0.01	0.11 ± 0.01	0.11 ± 0.01	0.10 ± 0.01	RI, MS
7	*β*-Pinene	983	978	0.47 ± 0.05	0.48 ± 0.06	0.64 ± 0.11	0.57 ± 0.17	0.32 ± 0.02	RI, MS
8	Myrcene	995	992	1.11 ± 0.13	1.04 ± 0.18	1.12 ± 0.17	1.26 ± 0.13	1.32 ± 0.15	RI, MS
9	3-Octanol	997	995	0.13 ± 0.08	0.02 ± 0.01	0.10 ± 0.10	0.15 ± 0.13	0.04 ± 0.02	RI, MS
10	*α*-Phellandrene	1010	1005	0.16 ± 0.01	0.17 ± 0.01	0.17 ± 0.01	0.17 ± 0.01	0.19 ± 0.02	RI, MS
11	*δ*-3-Carene	1015	1011	–	0.02 ± 0.01	0.03 ± 0.02	–	0.03 ± 0.02	RI, MS
12	*α*-Terpinene	1021	1019	1.20 ± 0.14	1.34 ± 0.44	1.17 ± 0.08	1.32 ± 0.20	0.92 ± 0.05	RI, MS
13	*p*-Cymene	1030	1025	6.46 ± 2.47	5.50 ± 1.54	9.44 ± 1.18	10.27 ± 0.7	5.93 ± 0.48	RI, MS
14	Limonene	1034	1030	0.42 ± 0.07	0.41 ± 0.16	0.58 ± 0.21	0.67 ± 0.09	0.42 ± 0.09	RI, MS
15	1,8-Cineole	1037	1035	0.10 ± 0.06	0.11 ± 0.06	0.08 ± 0.05	0.02 ± 0.01	0.12 ± 0.07	RI, MS
16	*Z*-*β*-Ocimene	1039	1039	0.40 ± 0.05	0.32 ± 0.13	0.46 ± 0.25	0.55 ± 0.13	0.41 ± 0.08	RI, MS
17	*E*-*β*-Ocimene	1050	1050	0.04 ± 0.02	0.06 ± 0.02	0.07 ± 0.01	0.08 ± 0.01	0.20 ± 0.06	RI, MS
18	*γ*-Terpinene	1063	1062	5.45 ± 0.77	6.16 ± 2.30	5.08 ± 0.34	5.57 ± 0.99	3.49 ± 0.23	RI, MS
19	*Z*-Sabinene hydrate	1071	1070	0.21 ± 0.01	0.11 ± 0.04	0.21 ± 0.02	0.19 ± 0.02	0.26 ± 0.01	RI, MS
20	*Z*-Linalool oxide	1076	1074	–	–	–	tr	0.09 ± 0.05	RI, MS
21	Terpinolene	1092	1089	0.18 ± 0.01	0.18 ± 0.02	0.20 ± 0.02	0.21 ± 0.02	0.28 ± 0.04	RI, MS
22	Linalool	1103	1100	0.90 ± 0.17	1.01 ± 0.55	1.13 ± 0.22	1.28 ± 0.54	8.88 ± 3.17	RI, MS
23	*E*-*γ*-Caryophyllene	1106	1106	0.06 ± 0.02	0.05 ± 0.03	0.07 ± 0.01	0.06 ± 0.02	0.11 ± 0.04	RI, MS
24	1-Octenyl-3-acetate	1111	1113	–	0.02 ± 0.01	tr	0.02 ± 0.01	–	RI, MS
25	*p*-Menth-2-en-1-ol	1124	1122	–	–	–	–	–	RI, MS
26	Allo-Ocimene	1131	1132	–	–	–	–	0.09 ± 0.04	RI, MS
27	1,3,8-*p*-Menthatriene	1133		–	–	–	–	–	MS
28	Borneol	1172	1171	–	0.02 ± 0.01	–	–	0.24 ± 0.11	RI, MS
29	*Z*-Linalool oxide (pyranoid)	1177	1173	–	tr	tr	0.02 ± 0.01	tr	RI, MS
30	4-Terpineol	1183	1179	0.20 ± 0.02	0.20 ± 0.07	0.18 ± 0.02	0.20 ± 0.01	0.17 ± 0.02	RI, MS
31	*p*-Cymenol-8	1188	1184	0.48 ± 0.04	0.44 ± 0.02	0.50 ± 0.04	0.54 ± 0.03	0.44 ± 0.02	RI, MS
32	*α*-Terpineol	1195	1190	0.54 ± 0.06	0.47 ± 0.18	0.63 ± 0.30	0.73 ± 0.17	0.47 ± 0.07	RI, MS
33	*Z*-Dihydro carvone	1202	1200	0.14 ± 0.01	0.12 ± 0.06	0.16 ± 0.02	0.14 ± 0.03	0.13 ± 0.01	RI, MS
34	Nerol	1234	1228	–	tr	0.03 ± 0.02	–	0.06 ± 0.01	RI, MS
35	Thymol methyl ether	1238	1237	0.08 ± 0.01	0.05 ± 0.01	0.13 ± 0.07	0.15 ± 0.06	0.13 ± 0.07	RI, MS
36	Carvacrol methyl ether	1248	1241	1.94 ± 0.78	1.87 ± 1.19	3.58 ± 0.88	2.90 ± 1.67	1.84 ± 0.39	RI, MS
37	Geraniol	1260	1263	0.02 ± 0.02	–	–	0.03 ± 0.02	tr	RI, MS
38	Geranial	1280	1273	–	–	–	–	0.02 ± 0.01	RI, MS
39	*p*-Thymol	1286		0.10 ± 0.05	0.18 ± 0.10	0.15 ± 0.03	0.17 ± 0.01	0.07 ± 0.01	MS
40	Thymol	1295	1295	10.24 ± 6.6	7.34 ± 4.00	8.72 ± 1.97	14.67 ± 7.8	12.28 ± 2.2	RI, MS
41	Carvacrol	1309	1305	60.26 ± 10.2	64.22 ± 7.5	54.17 ± 6.3	48.16 ± 6.7	55.10 ± 3.1	RI, MS
42	Thymol acetate	1358	1359	0.09 ± 0.07	0.05 ± 0.03	0.19 ± 0.13	0.21 ± 0.06	0.08 ± 0.02	RI, MS
43	Carvacrol acetate	1377	1368	0.66 ± 0.13	0.58 ± 0.39	1.39 ± 0.10	0.92 ± 0.45	0.55 ± 0.06	RI, MS
44	*β*-Bourbonene	1384	1378	–	–	–	–	–	RI, MS
45	*E*-Caryophyllene	1435	1427	1.95 ± 0.40	2.37 ± 0.40	1.85 ± 0.30	1.64 ± 0.29	1.38 ± 0.25	RI, MS
46	Aromadendrene	1455	1436	0.43 ± 0.09	0.29 ± 0.04	0.49 ± 0.05	0.40 ± 0.08	0.20 ± 0.05	RI, MS
47	*α*-Humullene	1469	1452	0.15 ± 0.02	0.15 ± 0.02	0.16 ± 0.01	0.15 ± 0.02	0.10 ± 0.01	RI, MS
48	9-epi-(*E*)-Caryophyllene	1477	1474	0.02 ± 0.02	–	0.05 ± 0.02	0.02 ± 0.02	–	RI, MS
49	*E*-*β*-Guaiene	1504	1498	–	–	–	–	–	RI, MS
50	Viridiflorene	1510	1505	0.31 ± 0.07	0.21 ± 0.01	0.35 ± 0.03	0.28 ± 0.08	0.15 ± 0.03	RI, MS
51	Spathulenol	1594	1578	0.44 ± 0.13	0.27 ± 0.05	0.63 ± 0.04	0.59 ± 0.11	0.28 ± 0.02	RI, MS
52	Isoaromadendrene epoxide	1597		0.11 ± 0.02	0.11 ± 0.01	0.14 ± 0.01	0.15 ± 0.03	0.10 ± 0.02	MS
53	Caryophyllene oxide	1601	1599	0.21 ± 0.06	0.17 ± 0.03	0.30 ± 0.05	0.28 ± 0.06	0.19 ± 0.03	RI, MS
54	Caryophylla-4(12),8(13)-dien-5 beta-ol	1654	1663	0.09 ± 0.02	0.07 ± 0.01	0.11 ± 0.04	0.12 ± 0.02	0.08 ± 0.01	RI, MS
55	14-Hydroxy-9-epi-(*E*)-caryophyllene	1673	1669	0.06 ± 0.04	0.07 ± 0.01	0.11 ± 0.01	0.11 ± 0.02	0.06 ± 0.02	RI, MS
56	Khusinol	1686	1674	0.08 ± 0.02	0.07 ± 0.01	0.11 ± 0.01	0.11 ± 0.02	0.07 ± 0.01	RI, MS
	Monoterpene hydrocarbons			18.98	18.77	23.66	24.59	15.56	
	Oxygenated monoterpens			76.17	76.77	71.25	70.35	80.93	
	Sesquiterpene hydrocarbons			2.92	3.09	2.97	2.55	1.94	
	Oxygenated sesquiterpenes			0.99	0.76	1.40	1.35	0.78	
	Essential oil content (%)			4.8 ± 0.47	5.62 ± 0.91	5.18 ± 0.58	4.96 ± 0.34	4.15 ± 0.44

**Table:** 

N	Components	RI^a^	RI^b^	Populations				
				Daarbast	Konar Siah	Darab	Haneshk	Methods
1	*α*-Thujene	933	929	0.40 ± 0.24	0.43 ± 0.25	0.36 ± 0.03	0.28 ± 0.17	RI, MS	
2	*α*-Pinene	940	939	3.89 ± 2.00	3.31 ± 2.50	2.62 ± 0.28	1.54 ± 0.95	RI, MS	
3	Camphene	955	954	0.19 ± 0.07	0.14 ± 0.07	0.15 ± 0.01	0.10 ± 0.03	RI, MS	
4	2,4(10)-Thujadiene	960	957	–	–	–	–	RI, MS	
5	1,3-Octanol	969	973	0.07 ± 0.01	0.15 ± 0.01	0.16 ± 0.24	0.08 ± 0.02	RI, MS	
6	Sabinene	979	977	0.11 ± 0.03	0.21 ± 0.10	0.10 ± 0.01	0.10 ± 0.02	RI, MS	
7	*β*-Pinene	983	978	0.65 ± 0.28	0.56 ± 0.30	0.51 ± 0.04	0.30 ± 0.13	RI, MS	
8	Myrcene	995	992	1.10 ± 0.27	1.15 ± 0.29	0.94 ± 0.11	2.34 ± 0.76	RI, MS	
9	3-Octanol	997	995	0.18 ± 0.10	0.13 ± 0.10	0.04 ± 0.01	0.16 ± 0.09	RI, MS	
10	*α*-Phellandrene	1010	1005	0.18 ± 0.01	0.18 ± 0.01	0.13 ± 0.01	0.34 ± 0.11	RI, MS	
11	*δ*-3-Carene	1015	1011	0.03 ± 0.01	0.02 ± 0.01	–	–	RI, MS	
12	*α*-Terpinene	1021	1019	1.15 ± 0.15	1.25 ± 0.25	1.04 ± 0.06	0.81 ± 0.41	RI, MS	
13	*p*-Cymene	1030	1025	6.34 ± 2.69	6.42 ± 2.21	13.96 ± 1.1	4.33 ± 2.76	RI, MS	
14	Limonene	1034	1030	0.58 ± 0.27	0.58 ± 0.27	0.61 ± 0.09	0.76 ± 0.13	RI, MS	
15	1,8-Cineole	1037	1035	0.06 ± 0.01	0.06 ± 0.01	0.07 ± 0.05	0.19 ± 0.16	RI, MS	
16	*Z*-*β*-Ocimene	1039	1039	0.52 ± 0.30	0.59 ± 0.33	0.45 ± 0.04	0.68 ± 0.30	RI, MS	
17	*E*-*β*-Ocimene	1050	1050	0.08 ± 0.02	0.09 ± 0.03	0.04 ± 0.02	0.94 ± 0.50	RI, MS	
18	*γ*-Terpinene	1063	1062	4.73 ± 0.97	4.79 ± 1.03	2.93 ± 0.24	2.60 ± 1.76	RI, MS	
19	*Z*-Sabinene hydrate	1071	1070	0.20 ± 0.04	0.25 ± 0.05	0.21 ± 0.01	0.33 ± 0.27	RI, MS	
20	*Z*-Linalool oxide	1076	1074	0.10 ± 0.01	0.04 ± 0.01	0.04 ± 0.02	0.45 ± 0.30	RI, MS	
21	Terpinolene	1092	1089	0.21 ± 0.04	0.23 ± 0.06	0.21 ± 0.03	0.78 ± 0.28	RI, MS	
22	Linalool	1103	1100	1.25 ± 0.70	1.73 ± 1.10	1.69 ± 0.97	37.65 ± 20.6	RI, MS	
23	*E*-*γ*-Caryophyllene	1106	1106	0.06 ± 0.02	0.09 ± 0.06	0.10 ± 0.04	0.93 ± 0.50	RI, MS	
24	1-Octenyl-3-acetate	1111	1113	0.03 ± 0.01	0.07 ± 0.06	–	0.11 ± 0.07	RI, MS	
25	*p*-Menth-2-en-1-ol	1124	1122	0.04 ± 0.03	tr	–	0.16 ± 0.10	RI, MS	
26	allo-Ocimene	1131	1132	–	–	–	0.38 ± 0.26	RI, MS	
27	1,3,8-*p*-Menthatriene	1133		–	–	–	0.10 ± 0.08	MS	
28	Borneol	1172	1171	–	–	–	0.99 ± 0.68	RI, MS	
29	*Z*-Linalool oxide (pyranoid)	1177	1173	–	tr	0.12 ± 0.02	0.13 ± 0.06	RI, MS	
30	4-Terpineol	1183	1179	0.19 ± 0.04	0.16 ± 0.05	0.18 ± 0.02	0.18 ± 0.05	RI, MS	
31	*p*-Cymenol-8	1188	1184	0.48 ± 0.07	0.48 ± 0.05	0.59 ± 0.03	0.29 ± 0.09	RI, MS	
32	*α*-Terpineol	1195	1190	0.72 ± 0.37	0.81 ± 0.40	0.58 ± 0.04	0.60 ± 0.09	RI, MS	
33	*Z*-Dihydro carvone	1202	1200	0.19 ± 0.04	0.17 ± 0.08	0.03 ± 0.02	0.21 ± 0.11	RI, MS	
34	Nerol	1234	1228	0.02 ± 0.01	–	0.02 ± 0.01	0.06 ± 0.04	RI, MS	
35	Thymol methyl ether	1238	1237	0.08 ± 0.01	0.10 ± 0.08	1.52 ± 0.12	0.59 ± 0.50	RI, MS	
36	Carvacrol methyl ether	1248	1241	3.71 ± 3.00	3.23 ± 3.00	1.62 ± 0.38	0.92 ± 0.72	RI, MS	
37	Geraniol	1260	1263	tr	0.02 ± 0.01	tr	0.60 ± 0.28	RI, MS	
38	Geranial	1280	1273	–	–	–	–	RI, MS	
39	*p*-Thymol	1286		0.09 ± 0.05	0.07 ± 0.01	0.22 ± 0.03	0.05 ± 0.04	MS	
40	Thymol	1295	1295	5.26 ± 3.85	8.65 ± 8.30	41.61 ± 4.14	17.55 ± 10.9	RI, MS	
41	Carvacrol	1309	1305	61.41 ± 13.3	58.29 ± 13.3	21.81 ± 4.91	15.74 ± 12.9	RI, MS	
42	Thymol acetate	1358	1359	0.10 ± 0.10	0.17 ± 0.13	0.75 ± 0.09	0.21 ± 0.13	RI, MS	
43	Carvacrol acetate	1377	1368	1.12 ± 0.61	1.45 ± 1.23	0.31 ± 0.09	0.20 ± 0.09	RI, MS	
44	*β*-Bourbonene	1384	1378	–	–	–	0.04 ± 0.03	RI, MS	
45	*E*-Caryophyllene	1435	1427	1.93 ± 0.62	1.59 ± 0.36	1.59 ± 0.38	1.71 ± 0.62	RI, MS	
46	Aromadendrene	1455	1436	0.47 ± 0.07	0.38 ± 0.16	0.24 ± 0.09	0.19 ± 0.11	RI, MS	
47	*α*-Humulene	1469	1452	0.14 ± 0.02	0.12 ± 0.03	0.11 ± 0.02	0.16 ± 0.04	RI, MS	
48	9-epi-(*E*)-Caryophyllene	1477	1474	0.03 ± 0.01	0.03 ± 0.03	–	tr	RI, MS	
49	*E*-*β*-Guaiene	1504	1498	0.02 ± 0.01	tr	–	–	RI, MS	
50	Viridiflorene	1510	1505	0.38 ± 0.13	0.32 ± 0.18	0.14 ± 0.05	0.36 ± 0.15	RI, MS	
51	Spathulenol	1594	1578	0.48 ± 0.24	0.44 ± 0.24	0.35 ± 0.05	0.77 ± 0.31	RI, MS	
52	Isoaromadendrene epoxide	1597		0.10 ± 0.03	0.09 ± 0.04	0.21 ± 0.02	0.27 ± 0.10	MS	
53	Caryophyllene oxide	1601	1599	0.21 ± 0.04	0.20 ± 0.07	0.35 ± 0.03	0.45 ± 0.13	RI, MS	
54	Caryophylla-4(12),8(13)-dien-5 beta-ol	1654	1663	0.09 ± 0.02	0.07 ± 0.05	0.12 ± 0.01	0.21 ± 0.06	RI, MS	
55	14-Hydroxy-9-epi-(*E*)-caryophyllene	1673	1669	0.07 ± 0.03	0.05 ± 0.04	0.13 ± 0.01	0.16 ± 0.06	RI, MS	
56	Khusinol	1686	1674	0.08 ± 0.02	0.06 ± 0.04	0.12 ± 0.01	0.17 ± 0.05	RI, MS	
	Monoterpene hydrocarbons			20.16	19.95	24.05	16.38		
	Oxygenated monoterpens			75.05	75.75	71.37	77.21		
	Sesquiterpene hydrocarbons			3.03	2.53	2.18	3.39		
	Oxygenated sesquiterpenes			1.03	0.91	1.28	2.03		
	Essential oil content (%)			5.58 ± 0.48	5.89 ± 0.24	3.3 ± 0.26	3.71 ± 0.41		

tr, trace < 0.02%.

a: RI, linear retention indices on HP-5MS column, experimentally determined using homologue series of n-alkanes.

b: Relative retention indices taken from Adams and NIST.

Methods: MS, by comparison of the mass spectrum with those of the computer mass libraries Adams and NIST.

The Pearson correlations indicated positive and negative significant correlations between phytochemical compounds. Carvacrol had been positively correlated with carvacrol acetate (r = 0.70), carvacrol methyl ether (r = 0.54), and negatively correlated with linalool (r = −0.69), thymol (r = −0.64) and limonene (r = −0.79) while thymol was in significant negative correlation with carvacrol (r = −0.64). Furthermore, linalool had a significant positive correlation with *E*-*β*-ocimene (r = 0.99), myrcene (r = 0.97), limonene (r = 0.72), *Z*-*β*-ocimene (r = 0.69) and a negative correlation with *α*-terpinene (r = −0.72), *γ*-terpinene (r = −0.70), carvacrol (r = −0.69), *p*-cymene (r = −0.61), carvacrol acetate (r = −0.56) and carvacrol methyl ether (r = −0.53).

**Figure 3:**
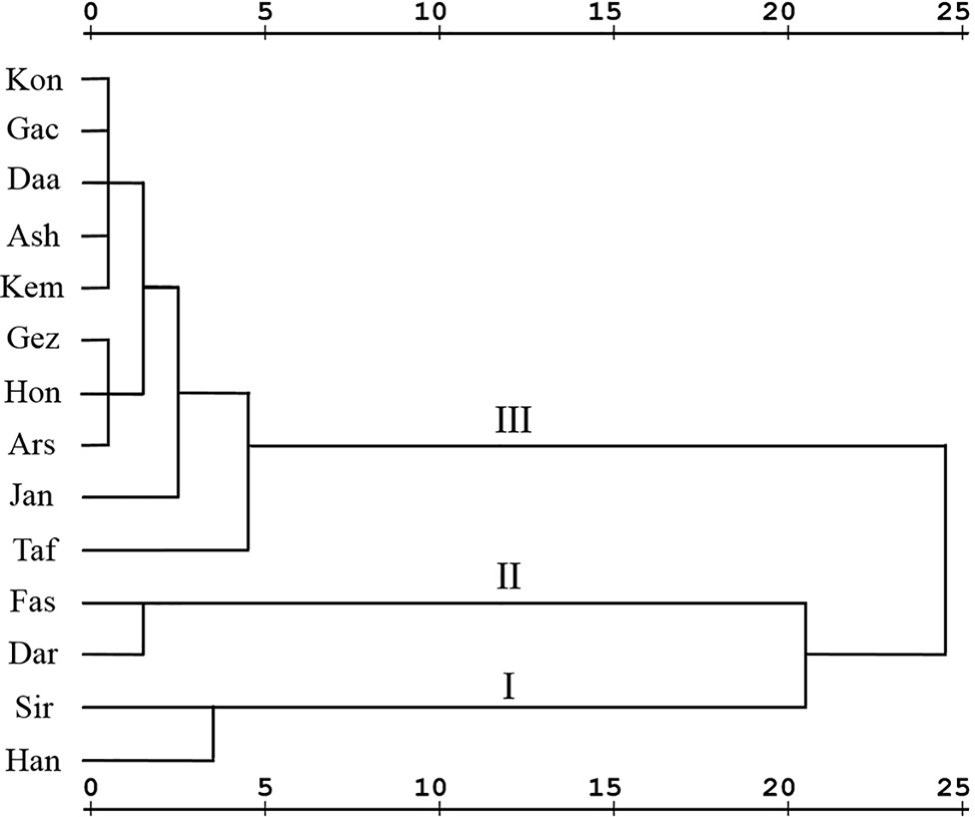
Hierarchical cluster analysis of *Zataria multiflora* populations based on phytochemical composition.

To determine the degree of phytochemical variation, HCA based on the phytochemical profiles was performed (**Figure 3**). According to the major components, three chemotypes can be distinguished thus populations of *Z. multiflora* were divided into three main clusters. Cluster I consists of two populations (Siriz and Haneshk) characterized by higher content of linalool. Cluster II contains two populations (Fasa and Darab) which are characterized by higher amounts of thymol, carvacrol, *p*-cymene, and linalool. Cluster III contains ten populations including Jandaq, Ashkezar, Taft, Arsenjan, Gezeh, Hongooyeh, Daarbast, Gachooyeh, Konar Siah, and Kemeshk characterized by lower quantities of *α*-pinene, myrcene, *α*-terpinene, linalool, and carvacrol methyl ether and higher amounts of carvacrol, thymol, *p*-cymene, and *γ*-terpinene.

### Environmental Characteristics

Geographical, climatic, and edaphic characteristics of *Z. multiflora* natural habitats are exhibited in **Tables 1** and **2**. Our results indicate that this species grows in areas characterized by a mean rainfall of 40.46 to 302.72 mm year^−1^ and mean annual temperature of 14.90°C to 28.80°C. The altitude ranges from 731 to 1946 m. The percentage of organic matter (OM) ranged from 4% to 10% (Haneshk and Darab regions, respectively). The soil of regions were rich in calcium (Ca), iron (Fe), potassium (K) and aluminum (Al) whereas nitrogen (N) and phosphor (P) were present in lower levels. Furthermore, *Z. multiflora* grows on soils with alkaline pH (7.60 to 7.90).

The volatile constituents were influenced by edaphic factors (**Table 4**). Carvacrol was significantly positively correlated with pH, Ca, and temperature [0.69 (p < 0.01), 0.62 and 0.54 (p < 0.05) respectively] and there was a highly negative correlation between carvacrol and Al, Fe, and K. The correlation analysis indicated that linalool was considerably positively correlated with Al, Fe, and K (p < 0.01). No statistically significant correlations were detected among N and EO content and phytochemical constituents.

**Table 4:** Pearson correlation coefficients between EO content, major components and edaphic factors.

Edaphic factors	EO	Linalool	Thymol	Carvacrol	*p*-Cymene
Altitude	−0.73**	0.53*	0.20	−0.41	−­0.45
Temperature	0.73**	−0.59*	−0.30	0.54*	0.37
OM	0.13	−0.55*	0.45	0.02	0.80**
pH	0.67**	−0.42	−0.64*	0.69**	0.01
Sand	0.22	0.41	−0.63*	0.15	−0.64*
Silt	−0.12	−0.56*	0.50	0.06	0.64*
Clay	−0.30	0.14	0.53*	−0.52	0.27
N	−0.31	−0.04	0.47	−0.27	0.49
P	−0.56*	0.44	0.04	−0.28	−0.23
K	−0.73**	0.57*	0.41	−0.63*	−0.25
Ca	0.62*	−0.65*	−0.29	0.62*	0.23
Fe	−0.78**	0.60*	0.50	−0.72**	−0.15
Al	−0.75**	0.55*	0.49	−0.68**	−0.17

Significance: *P < 0.05; **P < 0.01.

**Figure 4:**
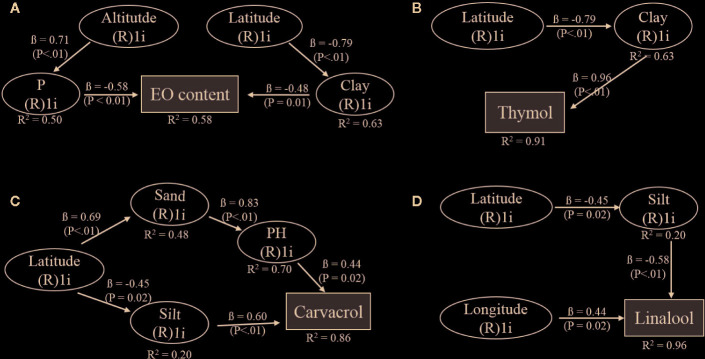
Hypothetical structural equation models (SEMs) to describe the relationships between geographical and edaphic factors and **(A)** EO, **(B)** thymol, **(C)** carvacrol, and **(D)** linalool content of *Zataria multiflora*. The climatic factors, temperature, and rainfall were included in the full model but did not explain EO or EO constituent content. R²: coefficient of determination indicating the variability explained for each variable. ß- values indicate the path coefficients, P: significance level for relationship.

The SEM approach was used to dissect the contribution of environmental factors on EO and EO constituent content. Significant SEMs for EO [APC = 0.641 (P < 0.001), ARS = 0.571 (P=0.002), AVIF = 1.001], thymol [APC = 0.874 (P < 0.001), ARS = 0.770 (P < 0.001), AVIF = 1.435], carvacrol [APC = 0.602 (P = 0.001), ARS = 0.560 (P = 0.002), AVIF = 1.536] and linalool [APC = 0.489 (P = 0.005), ARS = 0.655 (P=0.008), AVIF = 1.019] were obtained. The portion of clay and phosphor had a direct negative influence on EO content. The altitude had a positive effect on phosphor content while latitude had a negative effect on clay content in the soil (**Figure 4A**). Thymol content was positively affected by clay amount in the soil and indirect negatively via the negative effect of latitude on clay (**Figure 4B**). Carvacrol was directly positively influenced by silt content and pH-value in the soil, which was positively depended on the amount of sand in the soil (**Figure 4C**). Latitude had a negative effect on soil silt and a positive one on the soil sand portion. The linalool content was affected on the one hand, directly by longitude (positively) and on the other hand by silt (negatively) while silt content itself was negatively affected by latitude (**Figure 4D**).

### Quantitative Analysis of EO Composition by NIRS

**Figure 5:**
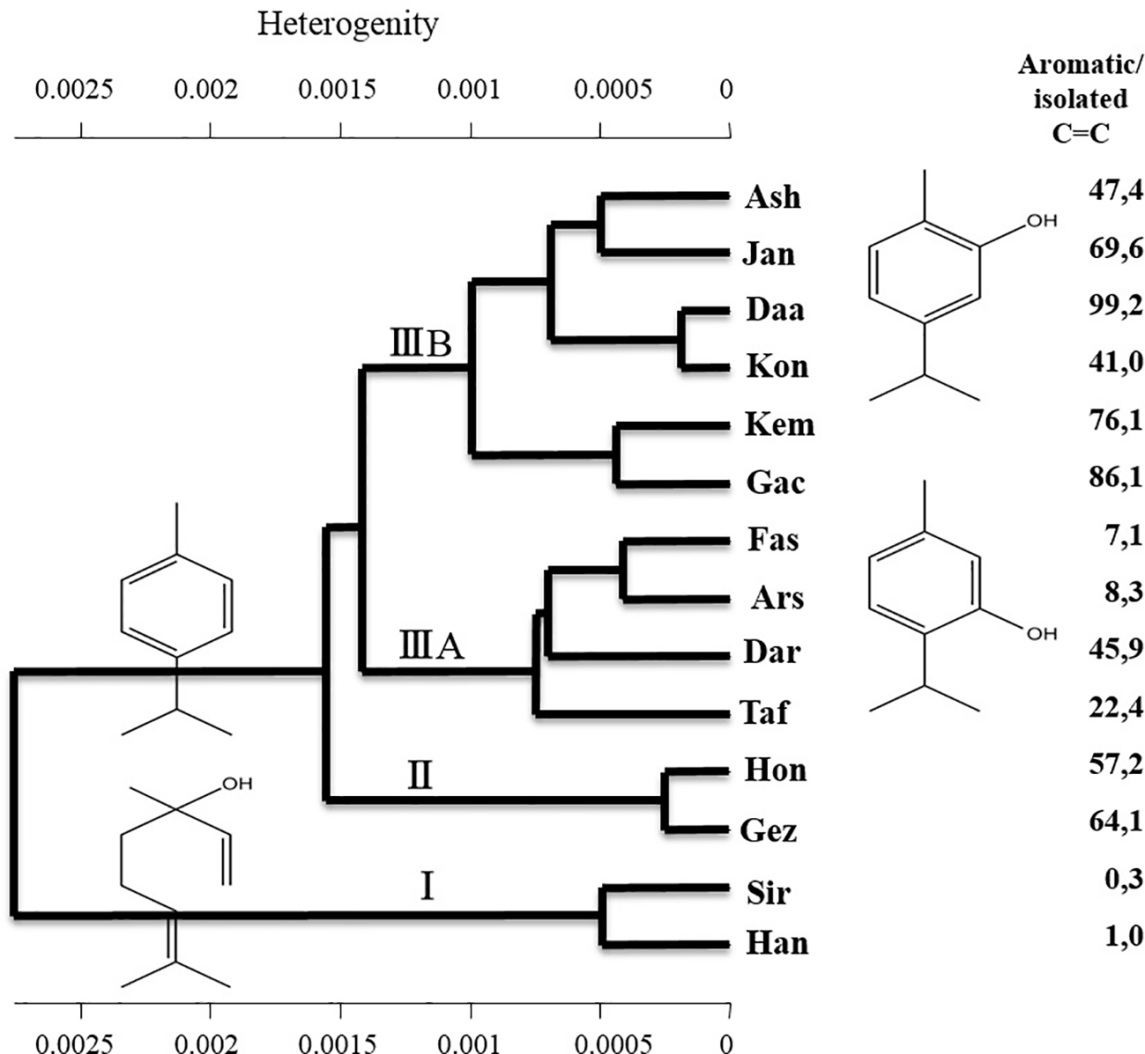
Hierarchical cluster analysis of the studied populations of *Zataria multiflora* based on the NIR spectra.

The dried leaves of specimens of *Z. multiflora* from different regions were analyzed by near infrared spectroscopy and hierarchical cluster analysis (HCA, Wards Algorithm). The NIR spectra of *Z. multiflora* were characterized by combination, first and second overtone vibrations in the range of 4,000 to 12,000 cm^−1^. HCA was used to group samples according to their spectral appearance determined through their chemical profile. **Figure 5** presents the appropriate HCA plot showing the separation of *Z. multiflora* populations into different clusters. In contrast to GC analysis, NIRS combines spectral features of chemically similar structures. Hence, carvacrol, thymol, and *p*-cymene, all characterized by an isopropyl- and methyl-substituted aromatic ring system, show all nearly identical NIRS absorption patterns. Therefore, for NIRS not only the quantity of individual EO components are relevant, but the amount of structurally related substances. As shown in **Figure 5** HCA resulted on highest level of heterogeneity in the clustering of samples according to the ratio of aromatic EO compounds (thymol + carvacrol + *p*-cymene) to aliphatic, isolated C=C structures (linalool). On the next level, types with a high content of aromatic structures are divided into sub-clusters with high amounts of carvacrol (cluster IIIB), high thymol, and high linalool or high *p*-cymene (cluster IIIA) or high carvacrol and high *p*-cymene (cluster II).

**Figure 6:**
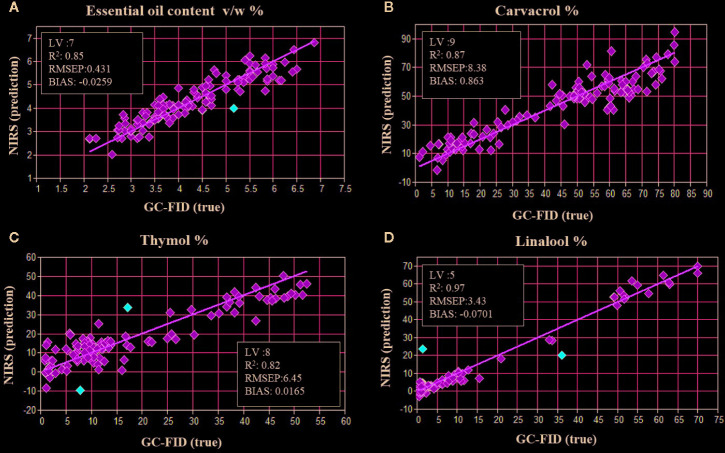
Results of 10-fold cross-validation of NIR and GC data for the **(A)** EO content, **(B)** carvacrol, **(C)** thymol, **(D)** linalool by correlation of averaged spectra for each population.

Chemometrics of superintended pattern identification based on PLS-DA of GC combined with NIR spectroscopy was endeavored to categorize fourteen populations of *Z. multiflora*. Quantification models for the EO content and for major compounds were developed by 10-fold cross-validation procedure according to literature (Krähmer et al., 2013). Therefore, averaged spectra for each plant were correlated with GC reference data for carvacrol, thymol, and linalool as well as EO content. For all constituents, appropriate prediction models were achieved. **Figure 6** shows the results of cross-validation according to plant wise averaged NIR spectra from all populations. Generally, coefficients of determination (R^2^) were higher than 0.82 for individual components and EO content. As shown in **Figure 6A**, NIRS offers a fast tool for estimation of EO content with a coefficient of determination R^2^ = 0.85 and a root mean square error of prediction (RMESP) below 10% of mean EO content (the mean of EO content over all samples used in the model, according to **Figure 6** something about 4 to 5 ml/100 g) (RMSEP = 0.431%). Furthermore, for major EO components, prediction quality was best for linalool (R^2^ = 0.97) followed by R^2^ = 0.87 and R^2^ = 0.82 for carvacrol and thymol (**Figures 6B–D**), respectively.

## Discussion

This study investigated the effect of different environmental factors on EO production, the content of specific EO compounds as well as on chemotype of different *Z. multiflora* populations. The EO values (up to 5.89% dry weight) detected in 14 populations in Iran were higher than those reported previously in the literature including 1.2% to 3.4% (Hadian et al., 2011a), 2.91% to 4% (Sadeghi et al., 2015), and 1.93% to 2.22% (Golkar et al., 2020). The EO content can be affected by geological, climatic, and edaphic characteristics as well as harvesting time. Saei-Dehkordi et al. (2010) described that the largest quantity of the EO content of *Z. multiflora* was collected in mid-May with 1.57% (v/w). Thus, knowledge on the season, phenological stage, and harvesting time during the day is necessary to obtain high quantities of EO content. Of the chemical constituents detected, carvacrol, thymol, linalool, *p*-cymene, *γ*-terpinene, and *α*-pinene were found as the main compounds of *Z. multiflora*. In other studies, the highest diversity was shown for the monoterpenes, including carvacrol, thymol, linalool, and *p*-cymene (Shafiee and Javidnia, 1997; Abkenar et al., 2008; Mahboubi and Bidgoli, 2010; Ziaee et al., 2018). Carvacrol, the major compound of the Jandagh population, has been previously reported as one of the most important components of EO among various members of the Lamiaceae family (Ebrahimi et al., 2008; Hadian et al., 2011b; Stefanaki et al., 2018; Santos et al., 2019). The main component of Darab and Fasa populations was thymol (41.61% and 48.12% respectively), which is an isomer of carvacrol. Saei-Dehkordi et al. (2010) and Sharififar et al. (2007) had depicted thymol as the most abundant component in the essential oil profile of *Z. multiflora* from different areas in Iran. Contrariwise, two other studies showed carvacrol as the main constituent of *Z. multiflora* (Basti et al., 2007; Khosravi et al., 2009). Moreover, EO of *Z. multiflora* contains other important monoterpene constituents like linalool, *p*-cymene, *γ*-terpinene, and *α*-pinene. Siriz and Haneshk populations were rich in linalool (55.38% and 37.65% respectively) and *p*-cymene was one of the main components of Darab population (13.96%).

The positive and negative correlations between EO components indicate the presence of three different chemotypes: thymol, carvacrol, and linalool. Furthermore, they indicate which compounds are interlinked in a chain of monoterpene synthesis with certain branches in the predicted enzymatic pathway: while geranyl-diphosphate is the precursor of non-phenolic linalool and phenolic thymol and carvacrol, the latter are connected *via p*-cymene (Thompson, 2005). In agreement to our results, similar correlations between individual EO components were found in *Artemisia dracunculus*, where methyl chavicol as the main constituent of *A. dracunculus* was positively correlated with terpinolene and methyl eugenol, and negatively correlated with *α*-pinene, limonene, (*Z*)-*β*-ocimene, and (*E*)-*β*-ocimene (Karimi et al., 2015). Hierarchical cluster analysis based on phytochemical components was proven to be a helpful tool to classify medicinal and aromatic plants accessions. For instance, cluster analysis on *Verbascum songaricum* resulted into nine groups (Selseleh et al., 2019) and for lemon balm populations three different chemotypes could be identified (Pouyanfar et al., 2018). Also grouping based on EO constituents of four *Vitex specimens* revealed different clusters (de Sena Filho et al., 2017). In the present study, the components of the EO measured at full flowering stage underpin the presence of the three chemotypes (carvacrol, thymol, linalool).

Rapid and reliable identification of medicinal plant species and chemotypes concerning authenticity and quality is crucial for pharmaceutical and food processing. Spectroscopy techniques as fast and easy handling technologies are nowadays widely applied directly on plant material for qualitative and semi-quantitative characterization. Different studies describe the application of NIRS, IRS, and Raman for differentiation of chemotypes and prediction of EO composition in various medicinal and aromatic plants (Seidler-Lozykowska et al., 2010; Gudi et al., 2014; Farag et al., 2018). For *Z. multiflora* the presented quantification models are not accurate for exact determination at current state, since, e.g., for linalool, samples are very inhomogeneous distributed over the investigated range of concentration. Nevertheless, in combination with HCA, near infrared spectroscopy offers a fast method for chemotyping and EO estimation already on plant material. An improved prediction of EO content and main components with regard to cross-validation concerning averaged ATR−FTIR spectra can also be achieved for constituents with lower concentrations (Gudi et al., 2015). The high correlation between NIRS and GC data allows application of NIRS for authenticity and quality control directly on the plant material for the flavor and fragrance as well as pharmaceutical industries. NIR spectroscopy can be used to classify plants according to their chemotype as well as predict the content of valuable components such as carvacrol, thymol, and linalool as well as other terpenes, rapidly and accurately.

The effect of soil parameters and climatic condition on plant perfomance and EO content has been shown for many plant species. For example, *Kelussia odoratissima* Mozaff grows in dark soil, rich in mineral content (Raiesi et al., 2013) and growth habitats of *Thymus pulegioides* were characterized by high amount of Al, Ca, Fe, K, and Si, however, by low amount of P and Mn (Vaičiulytė et al., 2017). Mexican oregano populations grown in soil with high nitrogen and iron content, lower soil water availability, and higher pH values showed a higher EO yield (Martínez-Natarén et al., 2012). It is widely accepted that environmental conditions affect plant EO content and its components (Ormeño et al., 2008; Mansour et al., 2010). Several studies have revealed that the predominance of carvacrol or thymol in different Lamiaceae species is related to environmental factors (Boira and Blanquer, 1998; Economou et al., 2011). In *Thymus vulgaris* such phenolic chemotypes cope better with summer drought, while non-phenolic (e.g., linalool) chemotypes cope better with early-winter freezing temperatures (Thompson et al., 2007). In our study the Pearson correlations revealed that altitude, K, Fe, and Al were significantly (p < 0.01) negatively correlated with EO content (**Table 3**). In agreement to our results, the lowest altitudes showed higher EO yield in *Lavandula angustifolia* (Demasi et al., 2018) and *Satureja rechingeri* (Hadian et al., 2014). Also, a correlation between higher EO yields at decreasing altitudes was found in *Origanum vulgare* (Giuliani et al., 2013). Notwithstanding the effect of geographical condition, EO content and EO constituents can be affected by edaphic factors and climatic conditions, for example, the soil type affects *Origanum syriacum* chemotype (El-Alam et al., 2019). In our study EO yield showed a highly significant positive correlation with temperature, pH value, and Ca. Former studies highlighted the same behavior in other aromatic plants and suggest that the wide variation in the chemical composition of the EO can be ascribed to habitat influences in *Origanum compactum* (Aboukhalid et al., 2017) and *Origanum vulgare* L. (De Falco et al., 2013). The influence of environmental conditions on EO of *Origanum vulgare* ssp. showed a negative correlation with altitude and a positive correlation with soil temperature and air temperature (Tuttolomondo et al., 2014). SEMs were applied to impute relationships between the different factors and revealed indirect geographic and direct edaphic effects on EO content and compounds, while climate factors do not have an influence. Chemotype and high amount of specific compounds can thus be predicted when looking for populations with specific features. Biotic factors like co-occuring vegetation (Wäschke et al., 2015) or herbivore activity (Dicke et al., 2009) can additionally influence the metabolome profile of plants and shall be considered in future studies.

## Conclusions

Medicinal and aromatic plants play important roles all over the world because of their wide application due to pharmacological, therapeutic, industrial, and agricultural properties. The varying climate and environmental growth conditions lead to a huge phytochemical diversity of these resources. *Zataria multiflora* is a valuable medicinal plant with various pharmaceutical properties and has potential as source of compounds with agricultural relevance as plant protection agents. Ingredients such as carvacrol, thymol, and linalool are responsible for the respective effects and show a high variability among the investigated populations. Environmental conditions are affecting the EO content and its components. Hence, existing variability in the chemical profile of studied populations allow selection of populations with distinct scent or bioactive components for use in pertinent industries and breeding purposes. Our approach of identifying environmental predictors for EO content, chemotype or presence of high amounts of specific compounds can help to identify regions for sampling plant material with the desired chemical profile. Based on mobile NIRS devices, fast classification of yet undescribed populations and individual plants together with an EO profiling can be performed directly in the field.

## Data Availability Statement

The datasets generated for this study are available on request to the corresponding authors.

## Author Contributions

TM, HS, and JH conceived and designed the project; AlK performed all sampling, extraction, and chemical analyses, except soil analysis which was performed by NH. Statistical analyses were performed by AlK, TM, and AnK. AlK and TM wrote the article with contributions from all other authors.

## Funding

The authors gratefully acknowledge the financial support obtained from the Federal Ministry of Food and Agriculture (BMEL) based on a decision of the Parliament of the Federal Republic of Germany *via* the Federal Office for Agriculture and Food (BLE) under the innovation support program (Project 2816DOKI06).

## Conflict of Interest

The authors declare that the research was conducted in the absence of any commercial or financial relationships that could be construed as a potential conflict of interest.

## References

[B1] Abkenar S. D. Yamini Y. Shemirani F. Assadi Y. (2008). Headspace solid phase microextraction using a porous-layer activated charcoal coating fused silica fiber for identification of volatile organic compounds emitted by *Zataria multiflora* Boiss. Chem. Anal-Warsaw 53, 277–287.

[B2] Aboukhalid K. Al Faiz C. Douaik A. Bakha M. Kursa K. Agacka-Mołdoch M. (2017). Influence of environmental factors on essential oil variability in *Origanum compactum* Bent. Chem. Biodivers. 14, e1700158. 10.1002/cbdv.201700158 28556574

[B3] Adams R. P. (2014). Identification of essential oil components by gas chromatography/mass spectrometry Vol. 456 (Carol Stream, IL: Allured publishing corporation).

[B4] Basti A. A. Misaghi A. Khaschabi D. (2007). Growth response and modelling of the effects of *Zataria multiflora* Boiss. essential oil, pH and temperature on *Salmonella typhimurium* and *Staphylococcus aureus* . LWT Food Sci. Technol. 40, 973–981. 10.1016/j.lwt.2006.07.007

[B5] Boira H. Blanquer A. (1998). Environmental factors affecting chemical variability of essential oils in *Thymus piperella* L. Biochem. Syst. Ecol. 26, 811–822. PII S0305-1978(98)00047-7. 10.1016/S0305-1978(98)00047-7

[B6] Chin W. W. Newsted P. R. (1999). Structural equation modeling analysis with small samples using partial least squares. Stat. Strateg. Small Sample Res. 1 (1), 307–341.

[B7] De Falco E. Roscigno G. Iodice C. Senatore F. (2013). Phytomorphological and Essential Oil Characterization *in situ* and ex situ of wild biotypes of *Oregano* collected in the Campania Region (Southern Italy). Chem. Biodivers. 10, 2078–2090. 10.1002/cbdv.201300185 24243616

[B8] de Sena Filho J. G. Barreto I. C. Soares Filho A. O. Nogueira P. C. Teodoro A. V. Cruz da Silva A. V. (2017). Volatile metabolomic composition of *Vitex* species: chemodiversity insights and acaricidal activity. Front. Plant Sci. 8, 1931. 10.3389/fpls.2017.01931 29184560 PMC5694497

[B9] Demasi S. Caser M. Lonati M. Cioni P. L. Pistelli L. Najar B. (2018). Latitude and altitude influence secondary metabolite production in peripheral alpine populations of the Mediterranean species *Lavandula angustifolia* Mill. Front. Plant Sci. 9, 983. 10.3389/fpls.2018.00983 30026754 PMC6042283

[B10] Dicke M. Van Loon J. J. Soler R. (2009). Chemical complexity of volatiles from plants induced by multiple attack. Nat. Chem. Biol. 5, 317. 10.1038/nchembio.169 19377458

[B11] Ebrahimi S. N. Hadian J. Mirjalili M. Sonboli A. Yousefzadi M. (2008). Essential oil composition and antibacterial activity of *Thymus caramanicus* at different phenological stages. Food Chem. 110, 927–931. 10.1016/j.foodchem.2008.02.083 26047281

[B12] Economou G. Panagopoulos G. Tarantilis P. Kalivas D. Kotoulas V. Travlos I. S. (2011). Variability in essential oil content and composition of *Origanum hirtum* L., *Origanum onites* L., *Coridothymus capitatus* (L.) and *Satureja thymbra* L. populations from the Greek island Ikaria. Ind. Crops Prod. 33, 236–241. 10.1016/j.indcrop.2010.10.021

[B13] El-Alam I. Zgheib R. Iriti M. El Beyrouthy M. Hattouny P. Verdin A. (2019). *Origanum syriacum* Essential Oil Chemical Polymorphism According to Soil Type. Foods 8, 90. 10.3390/foods8030090 PMC6463040 30841518

[B14] Farag N. F. El-Ahmady S. H. Abdelrahman E. H. Naumann A. Schulz H. Azzam S. M. (2018). Characterization of essential oils from Myrtaceae species using ATR-IR vibrational spectroscopy coupled to chemometrics. Ind. Crops Prod. 124, 870–877. 10.1016/j.indcrop.2018.07.066

[B15] Formisano C. Delfine S. Oliviero F. Tenore G. C. Rigano D. Senatore F. (2015). Correlation among environmental factors, chemical composition and antioxidative properties of essential oil and extracts of chamomile (*Matricaria chamomilla* L.) collected in Molise (South-central Italy). Ind. Crops Prod. 63, 256–263. 10.1016/j.indcrop.2014.09.042

[B16] Giuliani C. Maggi F. Papa F. Maleci Bini L. (2013). Congruence of phytochemical and morphological profiles along an altitudinal gradient in *Origanum vulgare* ssp. *vulgare* from Venetian Region (NE Italy). Chem. Biodivers. 10 (4), 569–583. 10.1002/cbdv.201300019 23576343

[B17] Golkar P. Mosavat N. Jalali S. A. H. (2020). Essential oils, chemical constituents, antioxidant, antibacterial and *in vitro* cytotoxic activity of different *Thymus* species and *Zataria multiflora* collected from Iran. S. Afr. J. Bot. 130, 250–258. 10.1016/j.sajb.2019.12.005

[B18] Gudi G. Krähmer A. Krüger H. Hennig L. Schulz H. (2014). Discrimination of fennel chemotypes applying IR and Raman spectroscopy: Discovery of a new *γ*-asarone chemotype. J. Agric. Food. Chem. 62, 3537–3547. 10.1021/jf405752x 24678882

[B19] Gudi G. Krähmer A. Krüger H. Schulz H. (2015). Attenuated total reflectance–Fourier transform infrared spectroscopy on intact dried leaves of sage (*Salvia officinalis* L.): accelerated chemotaxonomic discrimination and analysis of essential oil composition. J. Agric. Food. Chem. 63, 8743–8750. 10.1021/acs.jafc.5b03852 26360136

[B20] Hadian J. Ebrahimi S. N. Mirjalili M. H. Azizi A. Ranjbar H. Friedt W. (2011a). Chemical and genetic diversity of *Zataria multiflora* Boiss. accessions growing wild in Iran. Chem. Biodivers. 8, 176–188. 10.1002/cbdv.201000070 21259428

[B21] Hadian J. Hossein Mirjalili M. Reza Kanani M. Salehnia A. Ganjipoor P. (2011b). Phytochemical and morphological characterization of *Satureja khuzistanica* Jamzad populations from Iran. Chem. Biodivers. 8 (5), 902–915. 10.1002/cbdv.201000249 21560239

[B22] Hadian J. Esmaeili H. Nadjafi F. Khadivi-Khub A. (2014). Essential oil characterization of *Satureja rechingeri* in Iran. Ind. Crops Prod. 61, 403–409. 10.1016/j.indcrop.2014.07.034

[B23] Iranian Herbal Pharmacopoeia Committee (2002). Iranian Herbal Pharmacopoeia. (Tehran: Ministry of Health and Medical Education of Iran), 2, 40711.

[B24] Karimi A. Hadian J. Farzaneh M. Khadivi-Khub A. (2015). Phenotypic diversity and volatile composition of Iranian *Artemisia dracunculus* . Ind. Crops Prod. 65, 315–323. 10.1016/j.indcrop.2014.12.003

[B25] Khazdair M. R. Ghorani V. Alavinezhad A. Boskabady M. H. (2018). Pharmacological effects of *Zataria multiflora* Boiss L. and its constituents focus on their anti-inflammatory, antioxidant, and immunomodulatory effects. Fund. Clin. Pharmacol. 32, 26–50. 10.1111/fcp.12331 29125648

[B26] Khosravi A. R. Shokri H. Tootian Z. Alizadeh M. Yahyaraeyat R. (2009). Comparative efficacies of *Zataria multiflora* essential oil and itraconazole against disseminated *Candida albicans* infection in BALB/c mice. Braz. J. Microbiol. 40, 439–445. 10.1590/S1517-83822009000300003 24031384 PMC3768526

[B27] Kock N. Lynn G. (2012). Lateral collinearity and misleading results in variance-based SEM: An illustration and recommendations. JAIS 13 (7), 1–40. 10.17705/1jais.00302

[B28] Kock N. (2010). Using WarpPLS in e-collaboration studies: An overview of five main analysis steps. IJeC 6, 1–11. 10.4018/jec.2010100101

[B29] Krähmer A. Gudi G. Weiher N. Gierus M. Schütze W. Schulz H. (2013). Characterization and quantification of secondary metabolite profiles in leaves of red and white clover species by NIR and ATR-IR spectroscopy. Vib. Spectrosc. 68, 96–103. 10.1016/j.vibspec.2013.05.012

[B30] Mahboubi M. Bidgoli F. G. (2010). Antistaphylococcal activity of *Zataria multiflora* essential oil and its synergy with vancomycin. Phytomedicine 17, 548–550. 10.1016/j.phymed.2009.11.004 20171067

[B31] Mahboubi M. (2019). Therapeutic potential of *Zataria multiflora* Boiss. in treatment of irritable bowel syndrome (IBS). J. Diet. Suppl. 16, 119–128. 10.1080/19390211.2017.1409852 29333891

[B32] Mahmoudvand H. Mirbadie S. R. Sadooghian S. Harandi M. F. Jahanbakhsh S. Saedi Dezaki E. (2017). Chemical composition and scolicidal activity of *Zataria multiflora* Boiss essential oil. J. Essent. Oil Res. 29, 42–47. 10.1080/10412905.2016.1201546

[B33] Mansour A. Enayat K. Neda M. S. Behzad A. (2010). Antibacterial effect and physicochemical properties of essential oil of *Zataria multiflora* Boiss. Asian Pac. J. Trop. Med. 3 (6), 439–442. 10.1016/S1995-7645(10)60105-8

[B34] Martínez-Natarén D. A. Parra-Tabla V. Dzib G. Acosta-Arriola V. Canul-Puc K. A. Calvo-Irabién L. M. (2012). Essential oil yield variation within and among wild populations of Mexican oregano (*Lippia graveolens* HBK-Verbenaceae), and its relation to climatic and edaphic conditions. J. Essent. Oil-Bear Plants 15, 589–601. 10.1080/0972060X.2012.10644093

[B35] Milos M. Radonic A. Bezic N. Dunkic V. (2001). Localities and seasonal variations in the chemical composition of essential oils of *Satureja montana* L. and *S. cuneifolia* Ten. Flavour Fragr. J. 16, 157–160. 10.1002/ffj.965

[B36] Moazeni M. Larki S. Oryan A. Saharkhiz M. J. (2014). Preventive and therapeutic effects of *Zataria multiflora* methanolic extract on hydatid cyst: An *in vivo* study. Vet. Parasitol. 205, 107–112. 10.1016/j.vetpar.2014.07.006 25070528

[B37] Mohajeri F. A. Misaghi A. Gheisari H. Basti A. A. Amiri A. Ghalebi S. R. (2018). The effect of *Zataria multiflora* Boiss Essential oil on the growth and citrinin production of *Penicillium citrinum* in culture media and cheese. Food Chem. Toxicol. 118, 691–694. 10.1016/j.fct.2018.06.021 29908269

[B38] Morshedloo M. R. Salami S. A. Nazeri V. Maggi F. Craker L. (2018). Essential oil profile of oregano (*Origanum vulgare* L.) populations grown under similar soil and climate conditions. Ind. Crops Prod. 119, 183–190. 10.1016/j.indcrop.2018.03.049

[B39] Niczad A. Sharafzadeh S. Alizadeh A. Amiri B. Bazrafshan F. (2019). Variability in essential oil constituent, phenolic content, antioxidant and antimicrobial activities of different ecotypes of *Zataria multiflora* Boiss. from Iran. J. Essent. Oil Bear. Pl. 22, 1435–1449. 10.1080/0972060X.2020.1713221

[B40] NIST Chemistry WebBook, 69 (2002). NIST Standard Reference Database Number 69. Linstrom P. J. Mallard W. G. Eds. (National Institute of Standards and Technology, Gaithersburg, USA). Available at: http://webbook.nist.gov.

[B41] Ormeño E. Baldy V. Ballini C. Fernandez C. (2008). Production and diversity of volatile terpenes from plants on calcareous and siliceous soils: effect of soil nutrients. J. Chem. Ecol. 34, 1219. 10.1007/s10886-008-9515-2. 18670820

[B42] Pavela R. Žabka M. Vrchotová N. Tříska J. (2018). Effect of foliar nutrition on the essential oil yield of Thyme (Thymus vulgaris L.). Ind. Crops Prod. 112, 762–765. 10.1016/j.indcrop.2018.05.048

[B43] Pouyanfar E. Hadian J. Akbarzade M. Hatami M. Kanani M. R. Ghorbanpour M. (2018). Analysis of phytochemical and morphological variability in different wild-and agro-ecotypic populations of *Melissa officinalis* L. growing in northern habitats of Iran. Ind. Crops Prod. 112, 262–273. 10.1016/j.indcrop.2017.12.008

[B44] Raiesi S. Nadjafi F. Hadian J. Kanani M. R. Ayyari M. (2013). Autecological and phytochemical studies of Kelussia odoratissima Mozaff. an endangered ethnomedicinal plant of Iran. JBAPN 3, 285–294. 10.1080/22311866.2013.782748

[B45] Sadeghi H. Robati Z. Saharkhiz M. J. (2015). Variability in *Zataria multiflora* Bioss. essential oil of twelve populations from Fars province, Iran. Ind. Crops Prod. 67, 221–226. 10.1016/j.indcrop.2015.01.021

[B46] Saedi Dezaki E. Mahmoudvand H. Sharififar F. Fallahi S. Monzote L. Ezatkhah F. (2016). Chemical composition along with anti-leishmanial and cytotoxic activity of *Zataria multiflora* . Pharm. Biol. 54, 752–758. 10.3109/13880209.2015.1079223 26449681

[B47] Saei-Dehkordi S. S. Tajik H. Moradi M. Khalighi-Sigaroodi F. (2010). Chemical composition of essential oils in *Zataria multiflora* Boiss. from different parts of Iran and their radical scavenging and antimicrobial activity. Food Chem. Toxicol. 48, 1562–1567. 10.1016/j.fct.2010.03.025 20332011

[B48] Sajed H. Sahebkar A. Iranshahi M. (2013). *Zataria multiflora* Boiss. (Shirazi thyme) - an ancient condiment with modern pharmaceutical uses. J. Ethnopharmacol. 145, 686–698. 10.1016/j.jep.2012.12.018 23266333

[B49] Saleem M. Nazli R. Afza N. Sami A. Shaiq Ali M. (2004). Biological significance of essential oil of *Zataria multiflora* Boiss. Nat. Prod. Res. 18, 493–497. 10.1080/14786410310001608064 15595607

[B50] Santos J. D. Coelho E. Silva R. Passos C. P. Teixeira P. Henriques I. (2019). Chemical composition and antimicrobial activity of *Satureja montana* byproducts essential oils. Ind. Crops Prod. 137, 541–548. 10.1016/j.indcrop.2019.05.058

[B51] Schulz H. Baranska M. Belz H. H. Rösch P. Strehle M. A. Popp J. (2004). Chemotaxonomic characterisation of essential oil plants by vibrational spectroscopy measurements. Vib. Spectrosc. 35, 81–86. 10.1016/j.vibspec.2003.12.014

[B52] Schulz H. Özkan G. Baranska M. Krüger H. Özcan M. (2005). Characterisation of essential oil plants from Turkey by IR and Raman spectroscopy. Vib. Spectrosc. 39, 249–256. 10.1016/j.vibspec.2005.04.009

[B53] Seidler-Lozykowska K. Baranska M. Baranski R. Krol D. (2010). Raman analysis of caraway (*Carum carvi* L.) single fruits. Evaluation of essential oil content and its composition. J. Agric. Food. Chem. 58, 5271–5275. 10.1021/jf100298z 20402506

[B54] Selseleh M. Hadian J. Ebrahimi S. N. Sonboli A. Georgiev M. II Mirjalili M. H. (2019). Metabolic diversity and genetic association between wild populations of *Verbascum songaricum* (Scrophulariaceae). Ind. Crops Prod. 137, 112–125. 10.1016/j.indcrop.2019.03.069

[B55] Shafiee A. Javidnia K. (1997). Composition of essential oil of *Zataria multiflora* . Planta Med. 63, 371–372. 10.1055/s-2006-957707 17252397

[B56] Sharififar F. Moshafi M. H. Mansouri S. H. Khodashenas M. Khoshnoodi M. (2007). In vitro evaluation of antibacterial and antioxidant activities of the essential oil and methanol extract of endemic *Zataria multiflora* Boiss. Food Control 18, 800–805. 10.1016/j.foodcont.2006.04.002

[B57] Simbar M. Azarbad Z. Mojab F. Majd H. A. (2008). A comparative study of the therapeutic effects of the *Zataria multiflora* vaginal cream and metronidazole vaginal gel on bacterial vaginosis. Phytomedicine 15, 1025–1031. 10.1016/j.phymed.2008.08.004 18824338

[B58] Stefanaki A. Cook C. M. Lanaras T. Kokkini S. (2018). Essential oil variation of *Thymbra spicata* L. (Lamiaceae), an East Mediterranean “oregano” herb. Biochem. Syst. Ecol. 80, 63–69. 10.1016/j.bse.2018.06.006

[B59] Thompson J. D. Gauthier P. Amiot J. Ehlers B. K. Collin C. Fossat J. (2007). Ongoing adaptation to Mediterranean climate extremes in a chemically polymorphic plant. Ecol. Monogr. 77, 421–439. 10.1890/06-1973.1

[B60] Thompson J. D. (2005). Plant evolution in the Mediterranean (New York, United States: Oxford University Press Inc.).

[B61] Tuttolomondo T. Leto C. Leone R. Licata M. Virga G. Ruberto G. (2014). Essential oil characteristics of wild Sicilian oregano populations in relation to environmental conditions. J. Essent. Oil Res. 26, 210–220. 10.1080/10412905.2014.882278

[B62] Vaičiulytė V. Ložienė K. Taraškevičius R. Butkienė R. (2017). Variation of essential oil composition of *Thymus pulegioides* in relation to soil chemistry. Ind. Crops Prod. 95, 422–433. 10.1016/j.indcrop.2016.10.052

[B63] Wäschke N. Hancock C. Hilker M. Obermaier E. Meiners T. (2015). Does vegetation complexity affect host plant chemistry, and thus multitrophic interactions, in a human-altered landscape? Oecologia 179, 281–292. 10.1007/s00442-015-3347-x 25986560

[B64] Zgheib R. Chaillou S. Ouaini N. Kassouf A. Rutledge D. El Azzi D. (2016). Chemometric tools to highlight the variability of the chemical composition and yield of Lebanese *Origanum syriacum* L. essential oil. Chem. Biodivers. 13, 1326–1347. 10.1002/cbdv.201600061 27447100

[B65] Ziaee E. Razmjooei M. Shad E. Eskandari M. H. (2018). Antibacterial mechanisms of *Zataria multiflora* Boiss. essential oil against *Lactobacillus curvatus* . LWT 87, 406–412. 10.1016/j.lwt.2017.08.089

